# E-cigarette cessation interest and quit attempts among young adults reporting exclusive e-cigarette use or dual use with other tobacco products: How can we reach them?

**DOI:** 10.18332/tpc/172416

**Published:** 2023-11-16

**Authors:** Daisy Le, Annie C. Ciceron, Katelyn F. Romm, Michelle E. Clausen, Lorien C. Abroms, W. Douglas Evans, Amanda L. Graham, Carla J. Berg

**Affiliations:** 1 Department of Policy, Populations, and Systems, School of Nursing, George Washington University, Washington DC, United States; 2 Department of Prevention and Community Health, Milken Institute School of Public Health, George Washington University, Washington DC, United States; 3 George Washington Cancer Center, George Washington University, Washington DC, United States; 4 TSET Health Promotion Research Center, Stephenson Cancer Center, University of Oklahoma Health Sciences Center, OK, United States; 5 Department of Pediatrics, College of Medicine, University of Oklahoma Health Sciences Center, OK, United States; 6 Innovations Center, Truth Initiative, Washington DC, United States; 7 Department of Medicine, College of Medicine and Science, Mayo Clinic, Rochester, MN, United States

**Keywords:** e-cigarettes, vaping, young adults, perceptions, cessation

## Abstract

**INTRODUCTION:**

There is limited evidence to inform e-cigarette quitting interventions. This mixed-methods study examined: 1) e-cigarette and other tobacco product perceptions and cessation-related factors; and 2) potential behavioral intervention strategies among young adults reporting exclusive e-cigarette use or dual use with other tobacco products.

**METHODS:**

We analyzed Fall 2020 survey data from 726 participants reporting past 6-month e-cigarette use (mean age=24.15 years, 51.1% female, 38.5% racial/ethnic minority) from 6 US metropolitan areas and Spring 2021 qualitative interview data from a subset (n=40), comparing tobacco-related perceptions and cessation-related factors among those reporting exclusive use versus dual use.

**RESULTS:**

Among survey participants (35.5% exclusive e-cigarette use, 64.5% dual use), those reporting dual use indicated greater importance of quitting all tobacco or nicotine products (mean=5.28, SD=3.44 vs mean=4.65, SD=3.75, p=0.033), whereas those reporting exclusive use expressed greater confidence in quitting e-cigarettes (mean=7.59, SD=3.06 vs mean=7.08, SD=3.01, p=0.029) and all tobacco and nicotine products (mean=7.00, SD=3.16 vs mean=6.31, SD=3.13, p=0.008), as well as less favorable perceptions (i.e. more harmful to health and addictive, less socially acceptable) of cigarettes, cigars, and smokeless tobacco (p<0.05). Interview participants (50.0% exclusive e-cigarette use; 50.0% dual use) attributed previous failed e-cigarette quit attempts to their inability to cope with social influences, stress, and withdrawal symptoms. Although most expressed disinterest in quitting due to belief of eventually outgrowing e-cigarettes (among those reporting exclusive use) or unreadiness to abstain from nicotine (among those reporting dual use), many acknowledged the need for quitting interventions.

**CONCLUSIONS:**

Young adult e-cigarette cessation interventions should target risk perceptions, cessation barriers, and social influences/support.

## INTRODUCTION

E-cigarette use among young adults (aged 18–24 years) is a significant public health concern^1^ associated with other tobacco use and exposure to nicotine and hazardous chemicals^2,3^. In 2021, more than 2 million US young adults reported past 30-day use^2^. The short- and long-term health effects of e-cigarettes require targeted prevention efforts and cessation strategies^3^.

Although many young adults reporting e-cigarette use want to quit^4,5^, cessation services to support quit attempts are limited^6^. Efforts to advance e-cigarette cessation intervention research, have drawn upon previous smoking cessation research^4,6,7^ albeit lacking in substantive e-cigarette specific evidence^7^. Moreover, there are few large-scale intervention studies using experimental designs^8^. The existing literature includes only one large-scale, theory-based e-cigarette cessation intervention study for young adults – *This is Quitting* by Truth Initiative – a text messaging intervention for young people disseminated nationally through social media^4^. To date, >0.5 million young people have enrolled, and data from a large randomized trial among roughly 2600 young adults demonstrated the effectiveness of the program compared to control^9^.

Additional research is warranted to inform e-cigarette cessation efforts, including intervention targets and delivery strategies^6^. It is important to consider the complexities of social influences (i.e. how individuals adjust their behavior to meet the demands of a social environment), outcome expectations (i.e. anticipated, positive or negative, consequences as a result of engaging in a behavior), quit intentions, and dual use with other tobacco products among young adults reporting e-cigarette use^6,10–21^. As such, this mixed-methods study specifically sought to examine: 1) e-cigarette and other tobacco product perceptions and cessation-related factors that may serve as intervention targets; and 2) potential behavioral intervention strategies among young adults reporting exclusive e-cigarette use or dual use with other tobacco products.

## METHODS

### Study overview and design

This mixed-methods study analyzed Fall 2020 survey data and Spring 2021 interview data among young adults reporting e-cigarette use participating in a 2-year, 5-wave longitudinal cohort study, the Vape shop Advertising, Place characteristics and Effects Surveillance (VAPES) study^22^. The parent study, which was launched in Fall 2018, examined the US vape retail environment and its impact on use among young adults from 6 metropolitan statistical areas (MSAs: Atlanta, Boston, Minneapolis, Oklahoma City, San Diego, Seattle) selected based on differences in state tobacco control efforts.

### Quantitative data collection

Participants were recruited via ads on social media (Facebook, Reddit) targeting eligible individuals (i.e. aged 18–34 years, residing in one of the MSAs, English-speaking), using relevant headlines and imagery. Individuals who clicked on ads were directed to a webpage with a study description, consent form, and eligibility screener. Purposive, quota-based sampling ensured sufficient representation from those reporting e-cigarette use and individuals who smoke cigarettes (about 1/3 each), sexes, and racial/ethnic minorities. Subgroup enrollment was capped by MSA.

Overall, 65843 users of Facebook/Reddit viewed study ads, 10433 clicked ads, and 9847 consented. Of the 9847, only 2751 were either ineligible or excluded to reach subgroup target enrollment (1472 and 1279, respectively). Among the remaining, 3460 (48.8%) provided complete data and 3006 (86.9%) confirmed participation, after which they were enrolled and were emailed their first incentive ($10 e-gift card)^1^.

Quantitative analyses focused on W5 survey data (September–December 2020, n=2476; 82.4% of the 3006 at baseline) among those who reported past 6-month e-cigarette use (n=726).

### Measures

*Sociodemographics*

We included baseline age, gender, race/ethnicity, sexual orientation, education level, and relationship status.

*E-cigarette, tobacco, and marijuana use and related factors*

At Wave 5, participants reported days of e-cigarette use in the past 6 months; individuals reporting past 6-month use also reported days of use in the past 30. Similar items assessed use of cigarettes, hookah/waterpipe, little cigars/cigarillos, large cigars, smokeless tobacco, and cannabis.

Past 6-month individuals reporting e-cigarette use indicated the three flavors they most often use, e.g. tobacco, menthol or mint, fruit (Table 1); how often they used nicotine salt (i.e. nicotine base combined with acid(s) allowing nicotine to be inhaled more easily; 0=never, 5=all of the time)^2-5^; and device type used most often (disposable, closed cartridge-based [i.e. rechargeable closed system], pod/box mods [i.e. rechargeable open system], other).

**Table 1:** Sociodemographic characteristics and e-cigarette and other substance use behaviors among young adults in a cohort study who reported past 6-month e-cigarette use and participated in survey assessments in Fall 2020 (N=726) and/or who reported past 30-day e-cigarette use and participated in semi-structured interviews in Spring 2021 (N=40)

*Characteristics*	*Survey participants (N=726) n (%)*	*Interview participants (N=40) n (%)*
**Sociodemographics**		
**MSA**		
Atlanta	102 (14.0)	8 (20.0)
Boston	110 (15.2)	5 (12.5)
Minneapolis	134 (18.5)	10 (25.0)
Oklahoma City	77 (10.6)	3 (7.5)
San Diego	106 (14.6)	5 (12.5)
Seattle	131 (18.0)	6 (15.0)
Other	58 (8.0)	2 (5.0)
**Age** (years), mean (SD)	24.15 (4.84)	26.30 (4.39)
**Gender**		
Female	371 (51.1)	14 (35.0)
Male	333 (45.9)	25 (62.5)
Other	22 (3.0)	1 (2.5)
**Sexual minority**	258 (35.5)	18 (45.0)
**Race**		
White	534 (73.6)	23 (57.5)
Black	32 (4.4)	2 (5.0)
Asian	74 (10.2)	9 (22.5)
Other^a^	86 (11.8)	6 (15.0)
Hispanic	88 (12.1)	5 (12.5)
**Education level ≥Bachelor’s degree**	453 (62.4)	30 (75.0)
**Married/living with partner**	272 (37.5)	12 (30.0)
**E-cigarette use characteristics**		
**Flavors most commonly used** (list up to 3)		
Tobacco	98 (13.5)	8 (20.0)
Menthol or mint	356 (49.0)	23 (57.5)
Fruit flavors	502 (69.1)	33 (82.5)
Candy flavors	196 (27.0)	14 (35.0)
Caramel, vanilla, chocolate, cream	85 (11.7)	5 (12.5)
Coffee or tea	35 (4.8)	1 (2.5)
Alcohol drink flavors	26 (3.6)	0 (0)
Other	101 (13.9)	4 (10.0)
**Typically use nicotine salt**		
Never	290 (39.9)	14 (35.0)
Rarely	118 (16.3)	3 (7.5)
Some of the time	102 (14.0)	5 (12.5)
Most of the time	77 (10.6)	5 (12.5)
All of the time	139 (19.1)	13 (32.5)
**Device type**		
Disposable	156 (21.5)	4 (10.0)
Closed cartridge-based	188 (25.9)	12 (30.0)
Pod/box mods	347 (47.8)	23 (57.5)
Other	35 (4.8)	1 (2.5)
**Other tobacco/substance use^b^**		
Any other tobacco product use (i.e. dual-use)^c^	468 (64.5)	26 (65.0)
Cigarettes	380 (81.2)	24 (92.3)
Other tobacco products	248 (53.0)	15 (57.7)
Marijuana	486 (67.0)	25 (62.5)

a Other racial categories aggregated due to low frequency.

b 220 of those reporting past 6-month e-cigarette use reported only past 6-month cigarette use; 88 reported only past 6-month other tobacco product use (excluding cigarettes).

c Past 6-month use for survey participants, past 30-day use for interview participants.

*E-cigarette and other tobacco product perceptions*

To measure perceived risk (harm, addiction) and social acceptability, past 6-month individuals reporting e-cigarette use were asked: ‘How [harmful to your health/addictive/socially acceptable] do you think the following products are? e-cigarettes, cigarettes, cigar products, hookah, smokeless tobacco, marijuana’ (1=not at all to 7=extremely).

*Cessation-related factors*

To assess importance and confidence in quitting, they were asked: ‘How important is it that you quit using e-cigarettes?’ and ‘How confident are you that you could quit using e-cigarettes if you wanted to?’ (0=not at all to 10=absolutely). They were also asked: ‘Are you seriously thinking about quitting the use of e-cigarettes? Yes, within the next 30 days; Yes, within the next 6 months; Yes, in more than 6 months; or I am not thinking about quitting the use of e-cigarettes’. Participants were further asked: ‘During the past 12 months, how many times did you stop using e-cigarettes for one day or longer because you were trying to quit using e-cigarettes for good?’ and ‘Compared to a year ago, do you use e-cigarettes less, more, or about the same?’ They were also asked to answer questions 1–4 about quitting all tobacco or nicotine products.

### Qualitative data

In February–April 2021, W5 participants who reported past 30-day e-cigarette use were recruited via email to participate in a semi-structured interview, applying quota-based sampling to obtain sociodemographic diversity (i.e. sex, sexual orientation, race/ethnicity). Of those reporting past 30-day use of e-cigarettes who were recruited via email (n=139), 105 (75.5%) began the eligibility screener, of whom 94 (89.5%) completed the screener. Of the 94, 60 (63.8%) were eligible and consented. Of the 60, 40 (66.7%) completed an interview, at which point, saturation had been reached. The COREQ (Consolidated Criteria for Reporting Qualitative Research) guidelines were used to guide interview implementation and analyses^23^.

*Assessment*

The semi-structured interview guide explored experiences with tobacco product use and was initially piloted through pilot interviews among 4 graduate research assistants. Current analyses focused on e-cigarette risk perceptions, cessation interest, and potential intervention delivery strategies, for example:

- Which do you think is more harmful/addictive? Smoking cigarettes or vaping nicotine? Why?
- Have you heard of vaping-related injury, or how some people who vape have experienced negative respiratory symptoms? How concerned are you about this? Have you experienced symptoms?
- Are you interested in quitting vaping? How difficult do you think it would to quit? Have you ever tried?

For individuals reporting dual use with other tobacco products:

- How do you think your use with other tobacco products or marijuana would change if you stopped vaping?

Questions assessing potential behavioral intervention strategies included, for example:

- If you were to try to quit vaping, what information or messages would be helpful?
- What do you think are the most important motivators to quit?
- What in a vaping cessation program would be most helpful in convincing you to quit and/or to support you in successfully quitting?

Four trained female graduate research assistants facilitated Webex-based, audio-recorded interviews (about 45 minutes duration; compensation of $35 e-gift card), which were transcribed by a professional transcription service.

### Data analysis

Quantitative data were analyzed using descriptive statistics to characterize survey and interview participants. Bivariate analyses were conducted to compare e-cigarette and other tobacco product perceptions and cessation-related factors among past 6-month individuals reporting exclusive e-cigarette use versus dual use with other tobacco products. Analyses were conducted in SPSS 28.0, and alpha was set at 0.05.

Qualitative data were analyzed in NVivo 12 (QSR International) using thematic analysis methodology. Transcripts were systematically coded for patterns and recurring themes (as well as unusual or noteworthy themes) by a minimum of 2 coders and cross-checked for agreement regarding the application of the codes. Team discussions took place regularly in which the codebook themes were re-defined, inclusion and exclusion criteria set, and representative passages identified. Discrepancies regarding code choices were resolved through discussion in a process of constant comparison until consensus was reached (Kappa=93.3%). Interrater reliability was calculated for each code via intra-class correlation coefficients and deemed acceptable if ≥0.80. Content codes were used to thematically group similar interview text; themes were then organized into overarching domains based on use-profile (i.e. those reporting exclusive use of e-cigarettes versus dual use with other tobacco products) and compiled with representative quotations (edited for readability). Balancing the controversy in qualitative research regarding whether to quantify qualitative results, we chose to further indicate the frequency with which participants reported themes by ‘quantifying’ them as: ‘most’ (≥75%), ‘a majority’ (>50%, <75%), ‘many’ (nearly 50%), ‘some’ (≥20%), ‘several’ (<20%), and ‘a few’ (<10% of participants)^24,25^.

## RESULTS

### Quantitative results

In this sample of individuals reporting past 6-month e-cigarette use (n=726), average age was 24.15 years (SD=4.84), 51.1% were female, 35.5% sexual minority, 73.6% White, 4.4% Black, 10.2% Asian, and 12.1% Hispanic (Table 1). Among the 726 individuals reporting past 6-month use of e-cigarettes, 35.5% reported exclusive use of e-cigarettes and 64.5% reported dual use with other tobacco products. In terms of e-cigarette use characteristics, 84.8% used in the past 30 days, and the most used flavors were fruit (69.1%), menthol/mint (49.0%), and candy (27.0%). Additionally, 21.5% most often used disposable devices, 25.9% closed cartridge-based devices, and 47.8% pod/box mods; 43.7% used nicotine salt at least some of the time.

As shown in Table 2 *,* perceived harm to health and perceived addictiveness related to cigarettes, cigars, and smokeless tobacco products were greater among those reporting exclusive e-cigarette use versus dual use (all p<0.05); marijuana, however, was viewed as being more addictive among those reporting dual use (p=0.006). Those reporting exclusive use (vs dual use) also perceived cigarettes, cigars, and smokeless tobacco products as being less socially acceptable (all p<0.001).

**Table 2:** Factors related to e-cigarette cessation and all tobacco product cessation among young adults who participated in Fall 2020 survey assessments and reported past 6-month e-cigarette use (N=726)

*Variables*	*Exclusive e-cigarette use n (%)*	*Dual-use with other tobacco products n (%)*	*p*
**Total**	258 (35.5)	468 (64.5)	
**Perceived harm to health**, mean (SD)			
E-cigarettes	4.77 (1.66)	4.88 (1.67)	0.382
Cigarettes	6.80 (0.71)	6.42 (1.10)	**<0.001**
Cigar products	6.07 (1.21)	5.68 (1.51)	**<0.001**
Hookah	5.26 (1.64)	5.08 (1.75)	0.184
Smokeless tobacco	6.02 (1.23)	5.67 (1.52)	**0.001**
Marijuana	2.92 (1.50)	3.16 (1.71)	0.061
**Perceived addictiveness**, mean (SD)			
E-cigarettes	5.81 (1.65)	5.60 (1.61)	0.108
Cigarettes	6.51 (1.18)	6.09 (1.51)	**<0.001**
Cigar products	4.84 (1.77)	4.39 (1.86)	**0.002**
Hookah	4.30 (1.83)	4.03 (1.94)	0.073
Smokeless tobacco	5.70 (1.59)	5.22 (1.86)	**<0.001**
Marijuana	3.32 (1.81)	3.72 (1.89)	**0.006**
**Perceived social acceptability**, mean (SD)			
E-cigarettes	5.59 (1.59)	5.48 (1.57)	0.369
Cigarettes	3.32 (1.85)	4.11 (1.73)	**<0.001**
Cigar products	3.22 (1.69)	3.77 (1.57)	**<0.001**
Hookah	4.55 (1.90)	4.66 (1.73)	0.414
Smokeless tobacco	2.71 (1.79)	3.17 (1.88)	**0.001**
Marijuana	5.91 (1.67)	5.77 (1.68)	0.260
**E-cigarette use**, mean (SD)			
Importance quitting e-cigarettes	4.24 (3.67)	4.30 (3.55)	0.822
Confidence quitting e-cigarettes	7.59 (3.06)	7.08 (3.01)	**0.029**
**Readiness to quitting e-cigarettes**			0.906
Next 30 days	46 (17.8)	82 (17.5)	
Next 6 months	49 (19.0)	99 (21.2)	
>6 months	48 (18.6)	81 (17.3)	
Not thinking about quitting	115 (44.6)	206 (44.0)	
E-cigarette cessation attempt (past 12 months), mean (SD)	4.15 (9.08)	4.37 (8.30)	0.736
**E-cigarette use frequency compared to a year ago**			0.844
Less	94 (36.4)	161 (34.4)	
About the same	96 (37.2)	185 (39.5)	
More	52 (20.2)	98 (20.9)	
Didn’t use a year ago	16 (6.2)	24 (5.1)	
All tobacco product use*, mean (SD)			
Total, n	212	445	
Importance of quitting all tobacco or nicotine products	4.65 (3.75)	5.28 (3.44)	**0.033**
Confidence quitting all tobacco and nicotine products	7.00 (3.16)	6.31 (3.13)	**0.008**
Tobacco/nicotine cessation attempt (past 12 months)	2.98 (7.49)	3.73 (7.15)	0.215
**Readiness to quit all tobacco and nicotine products**			0.368
Next 30 days	21 (11.8)	61 (13.7)	
Next 6 months	45 (21.2)	94 (21.1)	
>6 months	43 (20.3)	111 (24.9)	
Not thinking about quitting	99 (46.7)	179 (40.2)	

* Assessed among those reporting past 30-day use of any tobacco product.

Regarding cessation-related factors, participants reporting dual use indicated greater importance of quitting all tobacco or nicotine products (mean=5.28, SD=3.44 vs mean=4.65, SD=3.75, p=0.033). However, those reporting exclusive e-cigarette use expressed greater confidence in quitting e-cigarettes (mean=7.59, SD=3.06 vs mean=7.08, SD=3.01, p=0.029) and all tobacco and nicotine products (mean=7.00, SD=3.16 vs mean=6.31, SD=3.13, p=0.008).

### Qualitative results

Semi-structured interview participants (n=40) were on average aged 26.30 years (SD=4.39), 35.0% female, 45.0% sexual minority, 57.5% White, 5.0% Black, 22.5% Asian, and 12.5% Hispanic (Table 1). The average number of days of e-cigarette use was 27.63 (SD=4.97). Among interview participants, 50% reported past 30-day use with other tobacco products (most commonly cigarettes) and 55.0% reported past 30-day use of marijuana. See Table 3 for themes, subthemes, and selected quotes related to qualitative findings below.

**Table 3:** Risk perceptions; cessation interest, history and barriers; and reactions to vaping cessation intervention strategies among young adults who participated in Spring 2021 semi-structured interviews and reported past 30-day exclusive e-cigarette use or co-use with other tobacco products (N=40)

	*Individuals reporting exclusive user of e-cigarettes+*	*Individuals reporting co-use with other tobacco products*
**E-cigarette and other tobacco product perception and cessation-related factors**		
**Risk perceptions**	**Smoking cigarettes more harmful than vaping due to combustion** I think smoking cigarettes just because it is combusting. It’s literally on fire. I don’t think vaping is necessarily good for you. I’m not gonna be one of those people. Because I mean, you are still taking a foreign object into your lungs. (Seattle, ever use of cigarettes and marijuana) I would say smoking cigarettes because of the combustion happening with the inhaling like actual smoke. I don’t think either is good for me, but I feel like I’d rather vape than get all the tar in my lungs and have teeth damage and all that. (Oklahoma City, current use of marijuana and ever use of cigarettes) I believe that smoking is way, way worse. And it’s because you’re literally lighting something on fire and inhaling direct carcinogens versus if you have a vape, that’s well maintained, the product is never getting to the point of being smoke, it’s vapor. So, it’s a different combination of things. I wouldn’t say it’s 100% safe, because you’re obviously putting something in your lungs that shouldn’t be there. But it is safer than putting smoke into your lungs. (San Diego, ever use of cigarettes and marijuana)	**Smoking cigarette more harmful than vaping nicotine** I think smoking cigarettes [is more harmful] … I’m automatically going [say] cigarette is worse because I’ve learned about all that since middle school. But I’m sure that vaping is similar. You’re just not thinking so much about it because of the pictures of cigarettes in the body just scares me. (Seattle, current use of cigarettes and marijuana) I just remember like all the advertisements from when I was a kid about how there’s like 17 cancer causing chemicals in cigarettes or whatever. And then also, like, people in my family have died because of lung cancer that they got from smoking cigarettes. (Oklahoma City, current use of cigarettes, other tobacco products, and marijuana)
**Interest in, prior attempts of, and barriers to vaping cessation**	No interest in vaping cessation now, but possibly in the future* As of right now, it’s like, I mean, unless I do get some debilitating health effect. That’ll require me to stop. I don’t really see any reason to stop. (Seattle, ever use of cigarettes and marijuana) No interest in vaping cessation* Oh, to be brutally honest. No, not really. It gets me through a day. It allows me a way to calm myself down at the end of the day. It’s better than going out and doing drugs and drinking and being a bum. (Minneapolis, no past/current use with other tobacco products or marijuana) **Health as a vaping cessation motivator** It’s changed over time, I really just want to quit nicotine altogether. I’ve tried like gummies and stuff in the past. They just sucked honestly. So like, ideally, smoking is just bad for you, regardless if it’s like regular cigarettes, e cigarettes, cigars, etc. So I know that much from time to time eliminate it altogether, for my health. (Boston, ever use of cigarettes and marijuana) **Unsuccessful vaping cessation attempt** I just decided to see if I can do it, and it didn’t really workout. (Minneapolis, no past/current use with other tobacco products or marijuana) Barriers overcoming the habit of vaping* Yeah, without finding something else to put in for vaping and smoking. I don’t want to drink to the point of being an alcoholic. … Drinking, drinking too much on a weekly basis is not going to make your body any better than smoking a cigarette. (Minneapolis, no past/current use with other tobacco products or marijuana) It goes back to the whole thing being bored. And my reasons for vaping. For me, it’s more of distracting myself from it. (SND, ever use of cigarettes and marijuana) Psychosocial triggers such as stress and anxiety as a cessation barrier* I’ve relied on the nicotine as a crutch to deal with my stress. So I think just learning to, to deal with that stress in a more healthy and productive way than just, you know, relying on going outside and vaping. … Stress definitely plays a huge factor in trying to convince my mind that I no longer need it. (Minneapolis, ever use of cigarettes, other tobacco products, and marijuana) **Withdrawal symptoms as a cessation barrier** The nicotine withdrawal would be the toughest. (Minneapolis, no past/current use with other tobacco products or marijuana)	Finances as a vaping cessation motivator* Definitely financial, because I can’t do both. There’s been times when I can, but right now with a job and all of that, I can do one or the other income wise. (Seattle, current use of cigarettes and marijuana) No interest in vaping cessation now, but possibly in the future* I need to cut out all nicotine products here. I’m trying to get by the end of the year, but we’ll see. (Seattle, current use of cigarettes and ever use with other tobacco products and marijuana) No interest in vaping cessation* I don’t really have a reason to. I could do it 100% if I wanted to, but I don’t want to. (Boston, current use of cigarettes and marijuana) Barriers overcoming the habit of vaping* Breaking the habit for sure. (Atlanta, current use of cigarettes and ever use of marijuana) Psychosocial triggers such as stress and anxiety as a cessation barrier* I would need a new coping mechanism for the stress. And then just the desire to not increase my cigarette intake. (Atlanta, current use of cigarettes and ever use of marijuana) **Social pressure as a cessation barrier** Definitely the social aspect of it. Just because, like, when everyone is like sitting around smoking, or vaping it’s kind of difficult to not take part in it. (Oklahoma City, current use of cigarettes and marijuana)
**Potential behavioral intervention strategies**		
**Message-related content and considerations**	**Information on science and health effects** I would say it all comes down to the necessity of wanting to live a longer, healthier life. When you start giving people the adverse health effects of what nicotine can do to the human body with you know, the tar in the nicotine that can turn your lungs black, you can have pockets formed, you can have crystallization of the longest, especially if you smoked menthols, like me. All these things can contribute to someone getting scared just enough to the point where they say I just don’t want to do it anymore. But again, you want to you want to base everything on facts, you don’t want to base anything just because of speculation. (Minneapolis, no past/current use with other tobacco products or marijuana) So, I already know how bad smoking is. If there was information on how vaping was bad that I think would seal the deal. (Minneapolis, no past/current use with other tobacco products or marijuana) The long-term effects on my health and stuff. (Seattle, no past/current use with other tobacco products or marijuana) Steps to quit and how to deal with withdrawal symptoms* I think these steps to quit will be more helpful than anything else. Um, because it’s sort of like, walks you through. How it must feel like to quit, so you’re better. Yeah, more prepared. (Minneapolis, no past/current use with other tobacco products or marijuana) With vaping, it would be to overcome the withdrawal symptoms and just that itch that your mind tells me like, I need to use this. (Boston, ever use of cigarettes and marijuana) So triggers, withdrawals, things like that. But I think … providing resources for that sort of thing would be beneficial … It’s difficult to find real material that’s helpful about quitting smoking. … We all know it’s bad, so providing resources that are like, this is how you can help deal withdrawals … [would be] so helpful. (Atlanta, ever use of cigarettes and marijuana) **Incentives and reward system** The effective parts are keeping yourself busy. … figuring out a different reward system, treating yourself in different ways changing routines. Like there’s, there’s a whole bunch of stuff you can do. (San Diego, ever use of cigarettes and marijuana) How to deal with stress and other emotional triggers* Stress, yeah, maybe like those coping mechanisms and things that would be beneficial? (Atlanta, ever use of cigarettes and current use of marijuana)	Information on science and health effects* I think it would have to be really about major health issue that [would make] me decide that I need to stop all that. (Atlanta, current use of cigarettes and marijuana) The risk or whatever is like well established, and cigarettes are bad for you, as published literature or e-cigs use for quitting, then I suppose we need more negative health data related studies that would be a strong motivator to quit. (Atlanta, current use of cigarettes) Steps to quit and how to deal with withdrawal symptoms* Some form of medication or alternative to kind of combat relaxing effect that nicotine has. And then also something to replace the like, urge to have something else kind of like it’s I do that a lot. Where I’ll just like have my juul like just kind of hanging out in my mouth without actually hitting it. (OKC, current use of cigarettes and marijuana) How to deal with stress and other emotional triggers* Maybe like stress, stress management techniques, potential infographics, and stuffs along that line. (Seattle, current use of cigarettes and ever use with other tobacco products and marijuana) Probably of ways to combat the emotional side effects of quitting. Or if there was information on other things to do that kind of mimic the effect of vaping, like habit wise. That would probably be helpful. (Oklahoma City, current use of cigarettes and marijuana)
**Intervention-related modality and delivery**	Interaction/support from coaches and/or peer counselors* I would say somebody else that’s vaping quit successfully. versus a doctor who’s going to give you the same, you know, the same line of like, you never should have started this. So clearly, you shouldn’t be doing this. It feels more condescending coming from a professional than it does from somebody who’s been there. (San Diego, ever use of cigarettes and marijuana) An than just having a person who’s either kind of going through the same thing, so you can like, kind of talk about what’s going on, and I’m bouncing ideas off of each other about how things are, how things are going, what things are working. So that way, you’re kind of like getting a little bit different ideas. (Minneapolis, ever use of cigarettes, other tobacco products, and marijuana)	Interaction/support from coaches and/or peer counselors* Someone has quit and has done it successfully, because that’s just going to be different than getting like medical doctors’ view. And, of course, they know everything. That’s right. So I like to hear more of like hearts. One, like a real story and seeing someone who has quit, that’s motivated, interesting, seeing an alcoholic who’s sober. It’s like, oh, okay, they could do it, I can do it kind of thing. (Oklahoma City, current use of cigarettes, other tobacco products, and marijuana) Text messaging as vehicle/medium of interaction with coaches/counselors* Probably over text … It’s more convenient. … If you’re in a social situation, you would be able to, like message them and talk to them about it without having everyone know. (Oklahoma City, current use of cigarettes and marijuana)

+ Inclusive of individuals reporting current use of marijuana.

* Similarities among individuals reporting exclusive user of e-cigarettes versus co-users with other tobacco products.

*E-cigarette and other tobacco product perceptions and cessation-related factors*

Almost all participants (regardless of dual use status) generally endorsed low perceived risks towards e-cigarette use; cigarettes were perceived as most harmful to health and more addictive than any amount of e-cigarette use. On the other hand, marijuana was generally regarded as less harmful than e-cigarettes:

*‘Vaping nicotine [is more harmful and addictive than marijuana] just because I’ve heard of a lot more health concerns about that online.’* (Minneapolis, current use of marijuana) and least likely to cause addiction across the various tobacco products.

Most individuals reporting exclusive use of e-cigarettes indicated that e-cigarettes were less detrimental than cigarettes due to the absence of combustion:

*‘The combustible [aspect] of proper cigarettes seems worse to me. … Just breathing something on fire versus something that’s just warmed up seems worse.’* (Seattle, ever use of cigarettes and marijuana)

and other harmful chemicals:

*‘I would say smoking cigarettes [is more harmful] because of the chemicals and heavy metals that are in there.’* (Atlanta, ever use of cigarettes and current use of marijuana)

However, those reporting dual use often described their risk perceptions of e-cigarettes within the context of other tobacco product use. For example, although some individuals reporting dual use were able to recognize that e-cigarettes can be as addictive as cigarettes, they perceived e-cigarettes as less harmful than cigarettes and other tobacco products:

*‘The nicotine itself [from vaping] is not the most dangerous aspect … smoking [cigarettes and other tobacco products] is much more harmful than [vaping] nicotine; [cigarettes and e-cigarettes], they’re both just as physically addictive. Vaping nicotine just makes me feel better. I have felt better since I’ve stopped smoking cigarettes ... cigarettes are more harmful.’* (Minneapolis, ever use of cigarettes and current use with other tobacco products and marijuana)

Interestingly, individuals reporting dual use shared varying perceptions on the health effects of e-cigarettes. Some disregarded any possible impact of e-cigarettes on their health outcomes due to the lack of research on the long-term effects of use:

*‘With vaping, I can at least live in ignorance; they haven’t done research into the long-term effects of vaping, from what I know.’* (Boston, current use of cigarettes and marijuana)

Yet, others clearly articulated concerns around the impact of use on their lungs and lung functioning:

*‘[Vaping causes] shortness of breath and chest pain. … I’ve been to the doctor a few times because of chest pain; I thought I was having a heart attack or had cancer.’* (Oklahoma City, current use of cigarettes, other tobacco products, and marijuana)

*‘There were some kids in my high school that got popcorn lung … which is not healthy.’* (Seattle, current use of cigarettes and marijuana)

When discussing their general interest in, prior attempts at, and barriers to e-cigarette cessation, participants reported a range of responses. While feelings of disinterest in immediate e-cigarette cessation were clearly expressed among most of the participants, the reasons behind the low interest differed across the user groups. Individuals reporting exclusive use of e-cigarettes felt confident that e-cigarettes were just going to be something that they would eventually:

*‘grow out of, [particularly] as less and less of [their] friends seem to be doing it.’* (Seattle, no past/current tobacco product or marijuana use)

They frequently expressed the lack of urgency to quit because use served as a temporary distraction:

*‘It goes back to the whole thing of me being bored - my reason for vaping. For me, it’s more about distracting myself [from the boredom].’* (San Diego, ever use of cigarettes and marijuana)

Potential negative social impact and changes were additional factors impacting interest e-cigarette cessation among individuals reporting exclusive use:

*‘When everyone is sitting around smoking or vaping, it’s kind of difficult to not take part in it.’* (Boston, ever use of cigarettes and current use of marijuana)

On the other hand, those reporting dual use commonly indicated that, while they would like to eventually cut out all forms of nicotine use, it was not something that they were quite ready for:

*‘Eventually, yes. But at the moment, not really. ... [Vaping] is not something I want to [do] forever because it is an addiction. Sometimes it feels like I don’t really have the choice in it. But at the moment, I’m kind of okay with that.’* (Oklahoma City, current use of cigarettes, other tobacco products, and marijuana)

However, some individuals reporting dual use shared that their awareness of the long-term health risks from e-cigarettes, such as lung injury, prompted quit attempts:

*‘I have heard of [“popcorn lungs”] and that was around the time whe[n] I started cutting down a lot [on vaping].’* (Seattle, current use of cigarettes and marijuana)

Individuals reporting exclusive use of e-cigarettes versus dual use with other tobacco products shared similar experiences regarding prior quit attempts or barriers to quitting. Both acknowledged that previous unsuccessful quit attempts were either due to their inability to deal with the day-to-day stressors:

*‘I've gone around two or three times, for two weeks without it. But in the end, I couldn’t give it up mainly because of like stressful factors, and [vaping] just helps me get through those times.’* (Boston, ever use of cigarettes and marijuana)

or inability to conveniently locate comparable replacements. Some participants noted withdrawal symptoms as a major barrier to cessation:

*‘I think [about] the chemical dependency piece in all this and how my body would react [if it] no longer [had] this like chemical, this drug, inside of it constantly … like what would that be like?’* (Seattle, ever use of cigarettes and current use of marijuana)

They indicated concern regarding whether quitting e-cigarettes would impact their other tobacco use, especially among individuals specifically looking for alternative coping:

*‘[I worry] that I won’t have a good alternative to [turn to, to deal with] the stress; that is the biggest challenge. I don’t have any concerns about withdrawal or anything, you know. Worst case scenario, I can just pick up a cigarette, right?’* (Atlanta, current use of cigarettes and ever use of marijuana)

While nearly all participants, regardless of dual use status, described e-cigarettes difficult to quit:

*‘I feel like it would be a lot more difficult to quit using e-cigs because I think I am probably a lot more addicted to them than cigarettes’*. (Minneapolis, ever use of cigarettes and current use of marijuana)

many also identified cost as a motivator for quitting:

*‘I just don’t want to spend as much on nicotine [to vape] anymore.’* (Seattle, ever use of cigarettes and current use of marijuana)

*Potential behavioral intervention strategies*

In terms of message-related content and considerations, most participants (regardless of dual use status) were interested in seeing side-by-side comparisons of various tobacco products and each product’s effect on one’s health:

*‘I’m really curious to know how it varies from cigarettes to vape. … I’d be really curious about what it’s like, to do one [product] versus [an]other.’* (Minneapolis, ever use of cigarettes and current use of marijuana)

Some suggested clearly sharing the evidence supporting messaging content:

*‘The same sort of information on nicotine usage [should be] unbiasedly compared [across tobacco products]. I would even be curious to see an honest comparison, on the [effects to one’s] health, [between] using marijuana versus nicotine or vaping marijuana versus smoking marijuana.’* (Minneapolis, ever use of cigarettes and current use with other tobacco products and marijuana)

While some individuals reporting dual use specified that e-cigarette cessation messaging should focus on coping and steps toward quitting, several individuals that reported exclusive use of e-cigarettes suggested a focus on the amount of nicotine consumed by using e-cigarettes; one participant mentioned:

*‘I’m constantly hitting it [so] I really use it way more. I don’t even know [how much], like compared to when I was smoking, I smoked a lot of cigarettes. I have no idea how much nicotine I’m consuming right now [when I vape].’* (Minneapolis, ever use of cigarettes and current use of marijuana)

With regard to intervention-related modality and delivery, preferences varied. While the majority (regardless of dual use status) indicated support of a text/web-based/app intervention as most useful for e-cigarette cessation:

*‘Probably just text, or a combination of text and email, like maybe a monthly check-in and the ability to text when needed.’* (San Diego, ever use of cigarettes and marijuana)

a few individuals reporting dual use opposed mobile-based resources, deeming them impersonal:

*‘That’s just something that feels very automated. I don’t think it would really [make] an impact, mentally. I would need to quit… but it would feel condescending.’* (Oklahoma City, current use of cigarettes, other tobacco products, and marijuana)

Another consideration for an effective e-cigarette cessation program for young adults, mentioned across both prompted and unprompted settings by many individuals reporting exclusive use of e-cigarettes and a majority of those reporting dual use, is the inclusion of peer coaches providing them ‘real time’ support via text messages *‘when needed’* (Minneapolis, ever use of cigarettes, other tobacco products, and marijuana); this would provide a more ‘convenient’ means for young adults to engage in cessation activities, as well as some *‘privacy’* during social situations when cessation support may be most needed (Boston, current use of cigarettes and marijuana). Most individuals reporting dual use also emphasized that the coach should be:

*‘an actual person that you’re talking to so that it would be more relatable and [not] feel so robotic.’* (Oklahoma City, current use of cigarettes, other tobacco products, and marijuana)

## DISCUSSION

This mixed-methods study revealed that young adults who reported using e-cigarettes generally held lower perceptions of risk associated with e-cigarette use in comparison to other tobacco products, particularly traditional cigarettes. Notably, irrespective of their dual use status, young adults who reported e-cigarettes use perceived e-cigarettes as posing the least harm to health among all tobacco and nicotine products, except for marijuana. Our qualitative findings yielded substantiating evidence and illuminated these quantitative results. The reduced risk perceptions can be attributed to several factors, including a lack of self-awareness regarding their own e-cigarette usage patterns and habits, especially when contrasted with the smoking intensity and inhalation associated with other tobacco product use. Additionally, beliefs related to e-cigarette addiction and a general lack of knowledge regarding the potential health consequences of e-cigarette use played a prominent role.

Many of our participants also expressed a lack of immediate interest in quitting e-cigarettes. Interestingly, those reporting dual use indicated greater importance of quitting all tobacco or nicotine products, yet they also exhibited lower confidence in achieving this goal compared to participants exclusively using e-cigarettes. Our interview data provided further insights into these perceptions: individuals reporting exclusive use of e-cigarettes were confident in eventually outgrowing this habit, while those reporting dual use recognized their current unpreparedness to abstain from all nicotine products.

Recognizing that risk perceptions are proximal predictors of e-cigarette use and cessation behavior, our study findings emphasize the critical necessity for researchers to purposefully address the low-risk perceptions of e-cigarettes among young adults, often linked to their limited knowledge of the potential health ramifications of e-cigarettes. For instance, participants frequently highlighted the importance of including information about the science and health effects of e-cigarettes^26^, strategies for managing withdrawal symptoms, and guidance on finding healthy and effective alternatives to e-cigarettes in intervention and message-related content.

Consistent with existing literature^6,27^, participants in both user groups emphasized the need for an effective behavioral intervention that not only provides strategies for overcoming the complex obstacles to cessation but also tailors messaging to accommodate their unique user profiles^6,27^. E-cigarette use among young adults is driven by diverse factors^28-30^, including peer influence^28-30^ and stress^28^. Those reporting exclusive use of e-cigarettes stressed the importance of addressing the social norms related to e-cigarettes, while those reporting dual use emphasized the need for specific coping strategies for daily stressors^21^. To enhance the relevance of messaging among individuals reporting exclusive use of e-cigarettes, intervention efforts should focus on reducing social cues associated with e-cigarette use and challenging the perceived social acceptability of e-cigarettes. For those reporting dual use, messages should explicitly target the challenges of coping with stress without resorting to e-cigarettes^21^ and should offer guidance on managing negative mood and withdrawal symptoms through techniques like relaxation training and physical activity. Key considerations to address cessation among individuals reporting dual use may also include highlighting the evidence regarding the health effects of dual use versus exclusively using e-cigarettes to discourage either new or continued cigarette smoking.

Finally, participants in both user groups expressed a desire for an ‘interactive’ program, potentially involving peer coaches (young adults who recently quit or are attempting to quit) providing ongoing cessation support either in-person or online. These findings align with the smoking cessation literature, which emphasizes the effectiveness of live coach/counselor-enhanced mobile health programs among young adults^31,32^. This further underscores the utility of such programs in reaching and engaging this population in vaping cessation. Given the complexity of e-cigarette use and its potential interactions with marijuana and other nicotine/tobacco products^3,21^, personalized counseling should also be considered to address the multifaceted challenges of cessation^12,33-35^.

### Limitations

Current findings are limited in their generalizability to other young adults due to our sampling strategy (e.g. recruiting roughly a third of individuals reporting past 30-day use of e-cigarettes and cigarettes) and attrition across waves of data collection. Participation in a longitudinal cohort study and across prior waves of data collection may have resulted in a unique sample. Additionally, data analyzed here are cross-sectional and only among individuals reporting current use of e-cigarettes, thus not capturing longitudinal associations or those reporting non-use, including those who may have quit using e-cigarettes by Wave 5 (Fall 2020), who differ relative to those reporting use^6^.

## CONCLUSIONS

Current findings emphasize the crucial demand for enhancing e-cigarette cessation interventions and messaging to accommodate the diverse needs, preferences, and usage patterns observed among different subgroups of individuals who report e-cigarette use. First and foremost, it is imperative to design targeted interventions that can adapt to the evolving needs of the varied profiles of young adults who report e-cigarette use, addressing their physical, mental, and emotional requirements. Additionally, these interventions should provide long-term support for individuals who require extended assistance. Secondly, recognizing the multifaceted needs of distinct groups of young adults reporting e-cigarette use, it becomes essential to offer multiple modes of delivery and engage peer coaches as interventionists who can provide real-time support. This approach holds the potential to increase quit rates significantly^36^. Lastly, ongoing surveillance of young adults’ responses to e-cigarette cessation intentions and barriers is necessary, particularly while considering their usage profiles, such as dual use. This is vital due to the limited existing research and the potential unintended consequences (e.g. individuals switching to potentially more harmful cigarettes) that could occur in the absence of clear messaging and accurate interpretation of intervention messages aimed at promoting e-cigarette cessation among young adults, especially those reporting dual use.

## Data Availability

The data supporting this research are available from the authors on reasonable request.
